# A Bivariate Chebyshev Spectral Collocation Quasilinearization Method for Nonlinear Evolution Parabolic Equations

**DOI:** 10.1155/2014/581987

**Published:** 2014-08-27

**Authors:** S. S. Motsa, V. M. Magagula, P. Sibanda

**Affiliations:** School of Mathematics, Statistics and Computer Science, University of KwaZulu-Natal, Private Bag X01, Scottsville, Pietermaritzburg 3209, South Africa

## Abstract

This paper presents a new method for solving higher order nonlinear evolution partial differential equations (NPDEs). The method combines quasilinearisation, the Chebyshev spectral collocation method, and bivariate Lagrange interpolation. In this paper, we use the method to solve several nonlinear evolution equations, such as the modified KdV-Burgers equation, highly nonlinear modified KdV equation, Fisher's equation, Burgers-Fisher equation, Burgers-Huxley equation, and the Fitzhugh-Nagumo equation. The results are compared with known exact analytical solutions from literature to confirm accuracy, convergence, and effectiveness of the method. There is congruence between the numerical results and the exact solutions to a high order of accuracy. Tables were generated to present the order of accuracy of the method; convergence graphs to verify convergence of the method and error graphs are presented to show the excellent agreement between the results from this study and the known results from literature.

## 1. Introduction

Nonlinearity exists everywhere and, in general, nature is nonlinear. Nonlinear evolution partial differential equations arise in many fields of science, particularly in physics, engineering, chemistry, finance, and biological systems. They are widely used to describe complex phenomena in various fields of sciences, such as wave propagation phenomena, fluid mechanics, plasma physics, quantum mechanics, nonlinear optics, solid state physics, chemical kinematics, physical chemistry, population dynamics, financial industry, and numerous areas of mathematical modeling. The development of both numerical and analytical methods for solving complicated, highly nonlinear evolution partial differential equations continues to be an area of interest to scientists whose research aim is to enrich deep understanding of such alluring nonlinear problems.

Innumerable number of methods for obtaining analytical and approximate solutions to nonlinear evolution equations have been proposed. Some of the analytical methods that have been used to solve evolution nonlinear partial differential equations include Adomian's decomposition method [1–3], homotopy analysis method [4–7], tanh-function method [8–10], Haar wavelet method [11–13], and Exp-function method [14–16]. Several numerical methods have been used to solve nonlinear evolution partial differential equations. These include the explicit-implicit method [17], Chebyshev finite difference methods [18], finite difference methods [19], finite element methods [20], and pseudospectral methods [21, 22].

Some drawbacks of approximate analytical methods include slow convergence, particularly for large time (*t* > 1). They may also be cumbersome to use as some involve manual integration of approximate series solutions and, hence, it is difficult to find closed solutions sometimes. On the other hand, some numerical methods may not work in some cases, for example, when the required solution has to be found near a singularity. Certain numerical methods, for example, finite differences require many grid points to achieve good accuracy and, hence, require a lot of computer memory and computational time. Conventional first-order finite difference methods may result in monotonic and stable solutions, but they are strongly dissipative causing the solution of the strongly convective partial differential equations to become smeared out and often grossly inaccurate. On the other hand, higher order difference methods are less dissipative but are prone to numerical instabilities.

Spectral methods have been used successfully in many different fields in sciences and engineering because of their ability to give accurate solutions of differential equations. Khater et al. [23] applied the Chebyshev spectral collocation method to solve Burgers type of equations in space and finite differences to approximate the time derivative. The Chebyshev spectral collocation method has been used together with the fourth-order Runge-Kutta method to solve the nonlinear PDEs in this study. The Chebyshev spectral collocation is first applied to the NPDE and this yields a system of ordinary differential equations, which are solved using the fourth-order Runge-Kutta method. Olmos and Shizgal [24], Javidi [25, 26], Dehghan and Fakhar-Izadi [27], Driscoll [28], and Driscoll [28] solved the Fisher, Burgers-Fisher, Burgers-Huxley, Fitzhugh-Nagumo, and KdV equations, respectively, using a combination of the Chebyshev spectral collocation method and fourth-order Runge-Kutta method. Darvishi et al. [29, 30] solved the KdV and the Burgers-Huxley equations using a combination of the Chebyshev spectral collocation method and Darvishi's preconditioning. Jacobs and Harley [31] and Tohidi and Kilicman [32] used spectral collocation directly for solving linear partial differential equations. Accuracy will be compromised if they implement their approach in solving nonlinear partial differential equations since they use Kronecker multiplication.

Chebyshev spectral methods are defined everywhere in the computational domain. Therefore, it is easy to get an accurate value of the function under consideration at any point of the domain, beside the collocation points. This property is often exploited, in particular to get a significant graphic representation of the solution, making the possible oscillations due to a wrong approximation of the derivative apparent. Spectral collocation methods are easy to implement and are adaptable to various problems, including variable coefficient and nonlinear differential equations. The error associated with the Chebyshev approximation is *O*(1/*N* ^*r*^) where *N* refers to the truncation and *r* is connected to the number of continuous derivatives of the function. The interest in using Chebyshev spectral methods in solving nonlinear PDEs stems from the fact that these methods require less grid points to achieve accurate results. They are computational and efficient compared to traditional methods like finite difference and finite element methods. Chebyshev spectral collocation method has been used in conjunction with additional methods which may have their own drawbacks. Here, we provide an alternative method that is not dependent on another method to approximate the solution.

The main objective of this work is to introduce a new method that uses Chebyshev spectral collocation, bivariate Lagrange interpolation polynomials together with quasilinearisation techniques. The nonlinear evolution equations are first linearized using the quasilinearisation method. The Chebyshev spectral collocation method with Lagrange interpolation polynomials are applied independently in space and time variables of the linearized evolution partial differential equation. This new method is termed bivariate interpolated spectral quasilinearisation method (BI-SQLM). We present the BI-SQLM algorithm in a general setting, where it can be used to solve any *r*th order nonlinear evolution equations. The applicability, accuracy, and reliability of the proposed BI-SQLM are confirmed by solving the modified KdV-Burger equation, highly nonlinear modified KdV equation, the Cahn-Hillard equation,the fourth-order KdV equation, Fisher's, Burgers-Fisher, Burger-Huxley, and the Fitzhugh-Nagumo equations. The results of the BI-SQLM are compared against known exact solutions that have been reported in the scientific literature. It is observed that the method achieves high accuracy with relatively fewer spatial grid points. It also converges fast to the exact solution and approximates the solution of the problem in a computationally efficient manner with simulations completed in fractions of a second in all cases. Tables are generated to show the order of accuracy of the method and time taken to compute the solutions. It is observed that, as the number of grid points is increased, the error decreases. Error graphs and graphs showing the excellent agreement of the exact and analytical solutions for all the nonlinear evolution equations are also presented.

The paper is organized as follows. In Section 2, we introduce the BI-SQLM algorithm for a general nonlinear evolution PDE. In Section 3, we describe the application of the BI-SQLM to selected test problems. The numerical simulations and results are presented in Section 4. Finally, we conclude in Section 5.

## 2. Bivariate Interpolated Spectral Quasilinearization Method (BI-SQLM)

In this section, we introduce the* Bivariate Interpolated Spectral Quasilinearization Method* (BI-SQLM) for finding solutions to nonlinear evolution PDEs. Without loss of generality, we consider nonlinear PDEs of the form

(1) ∂u∂τ=H(u,∂u∂η,∂2u∂η2,…,∂nu∂ηn),with  the  physical  region  τ∈[0,T], η∈[a,b],

where *n* is the order of differentiation, *u*(*η*, *τ*) is the required solution, and *H* is a nonlinear operator which contains all the spatial derivatives of *u*. The given physical region, *τ* ∈ [0, *T*], is converted to the region *t* ∈ [−1,1] using the linear transformation *τ* = *T*(*t* + 1)/2 and *η* ∈ [*a*, *b*] is converted to the region *x* ∈ [−1,1] using the linear transformation

(2) $$\begin{array}{c} \eta =\frac{1}{2}\left(b-a\right)x+\frac{1}{2}\left(b+a\right). \end{array}$$

Equation (1) can be expressed as

(3) $$\begin{array}{c} \frac{\partial u}{\partial t}=H\left(u,\frac{\partial u}{\partial x},\frac{\partial^{2}u}{\partial x^{2}},\ldots ,\frac{\partial^{n}u}{\partial x^{n}}\right),\;t\in \left[-1,1\right],\;\;x\in \left[-1,1\right]. \end{array}$$

The solution procedure assumes that the solution can be approximated by a bivariate Lagrange interpolation polynomial of the form

(4) $$\begin{array}{c} u\left(x,t\right)\approx \overset{N_{x}}{\underset{i=0}{\sum }}\;\overset{N_{t}}{\underset{j=0}{\sum }}u\left(x_{i},t_{j}\right)L_{i}\left(x\right)L_{j}\left(t\right), \end{array}$$

which interpolates *u*(*x*, *t*) at selected points in both the *x* and *t* directions defined by

(5) $$\begin{array}{c} \left\{x_{i}\right\}={\left\{\cos\left(\frac{\pi i}{N_{x}}\right)\right\}}_{i=0}^{N_{x}},\;\;\left\{t_{j}\right\}={\left\{\cos\left(\frac{\pi j}{N_{t}}\right)\right\}}_{j=0}^{N_{t}}. \end{array}$$

The choice of the Chebyshev-Gauss-Lobatto grid points (5) ensures that there is a simple conversion of the continuous derivatives, in both space and time, to discrete derivatives at the grid points. The functions *L*_*i*_(*x*) are the characteristic Lagrange cardinal polynomials

(6) $$\begin{array}{c} L_{i}\left(x\right)=\overset{N_{x}}{\underset{\begin{array}{c} i=0\\ i\neq k \end{array}}{\prod }}\frac{x-x_{k}}{x_{i}-x_{k}}, \end{array}$$

where

(7) $$\begin{array}{c} L_{i}\left(x_{k}\right)=\delta_{ik}=\{\begin{array}{cc} 0 & \text{if}\;i\neq k\\ 1 & \text{if}\;i=k. \end{array} \end{array}$$

The function *L*_*j*_(*t*) is defined in a similar manner. Before linearizing (3), it is convenient to split *H* into its linear and nonlinear components and rewrite the governing equation in the form

(8) $$\begin{array}{c} F\left[u,u^{\prime },\ldots ,u^{\left(n\right)}\right]+G\left[u,u^{\prime },\ldots ,u^{\left(n\right)}\right]-\dot{u}=0, \end{array}$$

where the dot and primes denote the time and space derivatives, respectively, *F* is a linear operator, and *G* is a nonlinear operator. Assuming that the difference *u*_*r*+1_ − *u*_*r*_ and all it's space derivative is small, we first approximate the nonlinear operator *G* using the linear terms of the Taylor series and, hence,

(9) G[u,u′,…,u(n)]≈G[ur,ur′,…,ur(n)] +∑k=0n∂G∂u(k)(ur+1(k)−ur(k)),

where *r* and *r* + 1 denote previous and current iterations, respectively. We remark that this quasilinearization method (QLM) approach is a generalisation of the Newton-Raphson method and was first proposed by Bellman and Kalaba [33] for solving nonlinear boundary value problems.

Equation (9) can be expressed as

(10) G[u,u′,…,u(n)]≈G[ur,ur′,…,ur(n)]+∑k=0nϕk,r[ur,ur′,…,ur(n)]ur+1(k)−∑k=0nϕk,r[ur,ur′,…,ur(n)]ur(k),

where

(11) $$\begin{array}{c} \phi_{k,r}\left[u_{r},u_{r}^{\prime },\ldots ,u_{r}^{\left(n\right)}\right]=\frac{\partial G}{\partial u^{\left(k\right)}}\left[u_{r},u_{r}^{\prime },\ldots ,u_{r}^{\left(n\right)}\right]. \end{array}$$

Substituting (10) into (8), we get

(12) F[ur+1,ur+1′,…,ur+1(n)]+∑k=0nϕk,rur+1(k)−u˙r+1 =Rr[ur,ur′,…,ur(n)],

where

(13) $$\begin{array}{c} R_{r}\left[u_{r},u_{r}^{\prime },\ldots ,u_{r}^{\left(n\right)}\right]=\overset{n}{\underset{k=0}{\sum }}\phi_{k,r}u_{r}^{\left(k\right)}-G\left[u_{r},u_{r}^{\prime },\ldots ,u_{r}^{\left(n\right)}\right]. \end{array}$$

A crucial step in the implementation of the solution procedure is the evaluation of the time derivative at the grid points *t*_*j*_ (*j* = 0,1,…, *N*_*t*_) and the spatial derivatives at the grid points *x*_*i*_ (*i* = 0,1,…, *N*_*x*_). The values of the time derivatives at the Chebyshev-Gauss-Lobatto points (*x*_*i*_, *t*_*j*_) are computed as (for *j* = 0,1, 2,…, *N*_*t*_)

(14) ∂u∂t|x=xi,t=tj=∑p=0Nx ∑k=0Ntu(xp,tk)Lp(xi)dLk(tj)dt=∑k=0Ntu(xi,tk)djk=∑k=0Ntdjku(xi,tk),

where *d*_*jk*_ = *dL*_*k*_(*t*_*j*_)/*dt* is the standard first derivative Chebyshev differentiation matrix of size (*N*_*t*_ + 1)×(*N*_*t*_ + 1) as defined in [34]. The values of the space derivatives at the Chebyshev-Gauss-Lobatto points (*x*_*i*_, *t*_*j*_)  (*i* = 0,1, 2,…, *N*_*x*_) are computed as

(15) ∂u∂x|x=xi,t=tj=∑p=0Nx ∑k=0Ntu(xp,tk)dLp(xi)dxLk(tj)=∑p=0Nxu(xp,tj)Dip=∑p=0NxDipu(xp,tj),

where *D*_*ip*_ = *dL*_*p*_(*x*_*i*_)/*dx* is the standard first derivative Chebyshev differentiation matrix of size (*N*_*x*_ + 1)×(*N*_*x*_ + 1). Similarly, for an *n*th order derivative, we have

(16) ∂nu∂xn|x=xi,t=tj=∑p=0NxDipnu(xp,tj)=DnUj,i=0,1,2,…,Nx,

where the vector **U**_*j*_ is defined as

(17) $$\begin{array}{c} U_{j}={\left[u_{j}\left(x_{0}\right),u_{j}\left(x_{1}\right),\ldots ,u_{j}\left(x_{N_{x}}\right)\right]}^{T} \end{array}$$

and the superscript *T* denotes matrix transpose. Substituting (16) into (12) we get

(18) F[Ur+1,j,Ur+1,j′,…,Ur+1,j(n)]+∑k=0nΦk,rUr+1,j(k)  −∑k=0NtdjkUr+1,k=Rr[Ur,j,Ur,j′,…,Ur,j(n)]

for *j* = 0,1, 2,…, *N*_*t*_, where

(19) $$\begin{array}{c} U_{r+1,j}^{\left(n\right)}=D^{n}U_{r+1,j},\\ \Phi_{k,r}=\left[\begin{array}{cccc} \phi_{k,r}\left(x_{0},t_{j}\right) & & & \\ & \phi_{k,r}\left(x_{1},t_{j}\right) & & \\ & & \ddots & \\ & & & \phi_{k,r}\left(x_{N_{x}},t_{j}\right) \end{array}\right]. \end{array}$$

The initial condition for (3) corresponds to *τ*_*N*_*t*__ = −1 and, hence, we express (18) as

(20) F[Ur+1,j,Ur+1,j′,…,Ur+1,j(n)]  +∑k=0nΦk,rUr+1,j(k)−∑k=0Nt−1djkUr+1,k=Rj,

where

(21) Rj=Rr[Ur,j,Ur,j′,…,Ur,j(n)]+djNtUNt,j=0,1,2,…,Nt−1.

Equation (20) can be expressed as the following *N*_*t*_(*N*_*x*_ + 1) × *N*_*t*_(*N*_*x*_ + 1) matrix system

(22) [A0,0A0,1⋯A0,Nt−1A1,0A1,1⋯A1,Nt−1⋮⋮⋱⋮ANt−1,0ANt−1,1⋯ANt−1,Nt−1][U0U1⋮UNt−1] =[R0R1⋮RNt−1],

where

(23) $$\begin{array}{c} A_{i,i}=F\left[I,D,\ldots ,D^{\left(n\right)}\right]+\overset{n}{\underset{k=0}{\sum }}\Phi_{k,r}D^{\left(k\right)}-d_{i,i}I,\\ A_{i,j}=-d_{i,j}I,\;\text{when}\;i\neq j, \end{array}$$

and **I** is the identity matrix of size (*N*_*x*_ + 1)×(*N*_*x*_ + 1). Solving (19) gives *u*(*x*_*i*_, *t*_*j*_) and, hence, we use (4) to approximate *u*(*x*, *t*).

## 3. Numerical Experiments

We apply the proposed algorithm to well-known nonlinear PDEs of the form (3) with exact solutions. In order to determine the level of accuracy of the BI-SQLM approximate solution, at a particular time level, in comparison with the exact solution, we report maximum error which is defined by

(24) $$\begin{array}{c} E_{N}=\underset{r}{\max}\left\{\left|u\left(x_{r},t\right)-\tilde{u}\left(x_{r},t\right)\right|,:0\leq r\leq N\right\}, \end{array}$$

where $\tilde{u}(x_{r},t)$ is the approximate solution and is the *u*(*x*_*r*_, *t*) exact solution at the time level *t*.

**Example 1 .**

We consider the generalized Burgers-Fisher equation [35]:

(25) $$\begin{array}{c} \frac{\partial u}{\partial t}+\alpha u^{\delta }\frac{\partial u}{\partial x}=\frac{\partial^{2}u}{\partial x^{2}}+\beta u\left(1-u^{\delta }\right), \end{array}$$

with initial condition

(26) $$\begin{array}{c} u\left(x,0\right)={\left\{\frac{1}{2}+\frac{1}{2}\tanh\left(\frac{-\alpha \delta }{2(\delta +1)}x\right)\right\}}^{1/\delta } \end{array}$$

and exact solution

(27) u(x,t) ={12+12tanh(−αδ2(δ+1)    ×[x−(αδ+1+β(δ+1)α)t])}1/δ,

where *α*, *β*, and *δ* are parameters. For illustration purposes, these parameters are chosen to be *α* = *β* = *δ* = 1 in this paper. The linear operator *F* and nonlinear operator *G* are chosen as

(28) $$\begin{array}{c} F\left(u\right)=u^{\prime \prime }+u,\;\;G\left(u\right)=-uu^{\prime }-u^{2}. \end{array}$$

We first linearize the nonlinear operator *G*. We approximate *G* using the equation

(29) $$\begin{array}{c} G\approx G\left[u_{r},u_{r}^{\prime },u_{r}^{\prime \prime }\right]+\overset{2}{\underset{k=0}{\sum }}\phi_{k,r}u_{r+1}^{\left(k\right)}-\overset{2}{\underset{k=0}{\sum }}\phi_{k,r}u_{r}^{\left(k\right)}. \end{array}$$

The coefficients are given by

(30) ϕ0,r=∂G∂u[ur,ur′,ur′′]=−(ur′+2ur),ϕ1,r=∂G∂u′[ur,ur′,ur′′]=−ur,ϕ2,r=∂G∂u′′[ur,ur′,ur′′]=0,Rr=∑k=02ϕk,rur(k)−G[ur,ur′,ur′′]=−ur2−urur′.

Therefore, the linearized equation can be expressed as

(31) $$\begin{array}{c} u_{r+1}^{\prime \prime }+\phi_{1,r}u_{r+1}^{\prime }+\phi_{0,r}u_{r+1}+u_{r+1}-\dot{u}=R_{r}. \end{array}$$

Applying the spectral method both in *x* and *t* and initial condition, we get

(32) D2Ur+1,i+Φ1,rDUr+1,i+Φ0,rUr+1,i  +Ur+1,i−2∑j=0Nt−1dijUr+1,j=Ri.

Equation (32) can be expressed as

(33) [A0,0A0,1⋯A0,Nt−1A1,0A1,1⋯A1,Nt−1⋮⋮⋱⋮ANt−1,0ANt−1,1⋯ANt−1,Nt−1][U0U1⋮UNt−1] =[R0R1⋮RNt−1],

where

(34) Ai,i=D2+Φ1,r(i)D+Φ0,r(i)+(1−2di,i)I,Ai,j=−2di,jI, when  i≠j,Ri=Rr+2diNtUr,Nt.

The boundary conditions are implemented in the first and last row of the matrices *A*_*ij*_ and the column vectors **R**_*i*_ for *i* = 0,1,…, *N*_*t*_ − 1 and *j* = 0,1,…, *N*_*t*_ − 1. The procedure for finding the variable coefficients *ϕ*_*i*_ and matrices for the remaining examples is similar.

**Example 2 .**

We consider Fisher's equation

(35) $$\begin{array}{c} \frac{\partial u}{\partial t}=\frac{\partial^{2}u}{\partial x^{2}}+\alpha u\left(1-u\right), \end{array}$$

subject to the initial condition

(36) $$\begin{array}{c} u\left(x,0\right)=\frac{1}{{\left(1+e^{\sqrt{\alpha /6}x}\right)}^{2}} \end{array}$$

and exact solution [36]

(37) $$\begin{array}{c} u\left(x,t\right)=\frac{1}{{\left(1+e^{\sqrt{\alpha /6}x-5\alpha t/6}\right)}^{2}}, \end{array}$$

where *α* is a constant. The Fisher equation represents a reactive-diffusive system and is encountered in chemical kinetics and population dynamics applications. For this example, the appropriate linear operator *F* and nonlinear operator *G* are chosen as

(38) $$\begin{array}{c} F\left(u\right)=u^{\prime \prime }+\alpha u,\;\;G\left(u\right)=-\alpha u^{2}. \end{array}$$

**Example 3 .**

Consider the Fitzhugh-Nagumo equation

(39) $$\begin{array}{c} \frac{\partial u}{\partial t}=\frac{\partial^{2}u}{\partial x^{2}}+u\left(u-\alpha \right)\left(1-u\right) \end{array}$$

with initial condition

(40) $$\begin{array}{c} u\left(x,0\right)=\frac{1}{2}\left[1-\coth\left(-\frac{x}{2\sqrt{2}}\right)\right]. \end{array}$$

This equation has the exact solution [37]

(41) $$\begin{array}{c} u\left(x,t\right)=\frac{1}{2}\left[1-\coth\left(-\frac{x}{2\sqrt{2}}+\frac{2\alpha -1}{4}t\right)\right], \end{array}$$

where *α* is a parameter. In this example, the linear operator *F* and nonlinear operator *G* are chosen as

(42) $$\begin{array}{c} F\left(u\right)=u^{\prime \prime }-\alpha u,\;\;G\left(u\right)=\left(1+\alpha \right)u^{2}-u^{3}. \end{array}$$

**Example 4 .**

Consider the Burgers-Huxley equation

(43) $$\begin{array}{c} \frac{\partial u}{\partial t}+\alpha u^{\delta }u_{x}=\frac{\partial^{2}u}{\partial x^{2}}+\beta u\left(1-u^{\delta }\right)\left(u^{\delta }-\gamma \right), \end{array}$$

where *α*, *β* ≥ 0 are constant parameters, *δ* is a positive integer (set to be *δ* = 1 in this study), and *γ* ∈ (0,1). The exact solution subject to the initial condition

(44) $$\begin{array}{c} u\left(x,0\right)=\frac{1}{2}-\frac{1}{2}\tanh\left[\frac{\beta }{r-\alpha }x\right], \end{array}$$

is reported in [38, 39] as

(45) $$\begin{array}{c} u\left(x,t\right)=\frac{1}{2}-\frac{1}{2}\tanh\left[\frac{\beta }{r-\alpha }\left(x-ct\right)\right], \end{array}$$

where

(46) $$\begin{array}{c} r=\sqrt{\alpha^{2}+8\beta },\;\;c=\frac{\left(\alpha -r\right)\left(2\gamma -1\right)+2\alpha }{4} \end{array}$$

The general solution (45) was reported in [40, 41]. In this example, the linear operator *F* and nonlinear operator *G* are chosen as

(47) $$\begin{array}{c} F\left(u\right)=u^{\prime \prime }-\beta \gamma u,\\ G\left(u\right)=-\alpha uu^{\prime }+\beta \left(1+\gamma \right)u^{2}-\beta u^{3}. \end{array}$$

**Example 5 .**

We consider the modified KdV-Burgers equation

(48) $$\begin{array}{c} \frac{\partial u}{\partial t}=\frac{\partial^{3}u}{\partial x^{3}}-\frac{\partial^{2}u}{\partial x^{2}}-6u^{2}\frac{\partial u}{\partial x} \end{array}$$

subject to the initial condition

(49) $$\begin{array}{c} u\left(x,0\right)=\frac{1}{6}+\frac{1}{6}\tanh\left(\frac{x}{6}\right) \end{array}$$

and exact solution [42]

(50) $$\begin{array}{c} u\left(x,t\right)=\frac{1}{6}+\frac{1}{6}\tanh\left(\frac{x}{6}-\frac{t}{27}\right). \end{array}$$

The modified KdV-Burgers equation describes various kinds of phenomena such as a mathematical model of turbulence [43] and the approximate theory of flow through a shock wave traveling in viscous fluid [44]. For this example, the linear operator *F* and nonlinear operator *G* are chosen as

(51) $$\begin{array}{c} F\left(u\right)=u^{\prime \prime \prime }-u^{\prime \prime },\;\;G\left(u\right)=-6u^{\prime }u^{2}. \end{array}$$

**Example 6 .**

We consider the high nonlinear modified KdV equation

(52) $$\begin{array}{c} \frac{\partial u}{\partial t}=\frac{\partial^{3}u}{\partial x^{3}}+{\left(\frac{\partial u}{\partial x}\right)}^{2}-u^{2} \end{array}$$

subject to the initial condition

(53) $$\begin{array}{c} u\left(x,0\right)=\frac{1}{2}+\frac{e^{-x}}{4} \end{array}$$

and exact solution

(54) $$\begin{array}{c} u\left(x,t\right)=\frac{1}{t+2}+\frac{e^{-(x+t)}}{{\left(t+2\right)}^{2}}. \end{array}$$

For this example, the linear operator *F* and nonlinear operator *G* are chosen as

(55) $$\begin{array}{c} F\left(u\right)=u^{\prime \prime \prime },\;\;G\left(u\right)={\left(u^{\prime }\right)}^{2}-u^{2}. \end{array}$$

## 4. Results and Discussion

In this section we present the numerical solutions obtained using the BI-SQLM algorithm. The number of collocation points in the space *x* variable used to generate the results is *N*_*x*_ = 10 in all cases. Similarly, the number of collocation points in the time *t* variable used is *N*_*t*_ = 10 in all cases. It was found that sufficient accuracy was achieved using these values in all numerical simulations.

In Tables 1, 2, 3, 4, 5, and 6 we give the maximum errors between the exact and BI-SQLM results for the Fisher equation, Burgers-Fisher equation, Fitzhugh-Nagumo equation, Burgers-Huxley equation, the modified KdV-Burgers equation, and the modified KdV equation, respectively, at *t* ∈ [0.1,1]. The results were computed in the space domain *x* ∈ [0,1]. To give a sense of the computational efficiency of the method, the computational time to generate the results is also given. Tables 1–6 clearly show the accuracy of the method. The accuracy is seen to improve with an increase in the number of collocation points *N*_*x*_. It is remarkable to note that accurate results with errors of order up to 10^−14^ are obtained using very few collocation points in both the *x* and *t* variables *N*_*t*_ ≤ 10, *N*_*x*_ ≤ 10. This is a clear indication that the BI-SQLM is powerful method that is appropriate in solving nonlinear evolution PDEs. We remark, also, that the BI-SQLM is computationally fast as accurate results are generated in a fraction of a second in all the examples considered in this work.

In Tables 7, 8, 9, 10, 11, and 12 we give the maximum errors of the BI-SQLM results for the Fisher equation, Burgers-Fisher equation, Fitzhugh-Nagumo equation, Burgers-Huxley equation, the modified KdV-Burgers equation, and the modified KdV equation, respectively, at selected values of *t* = 2 for different collocation points, *N*_*t*_, in the *t*-variable. The results in Tables 7–12 were computed on the space domain *x* ∈ [0,1]. We note that the accuracy does not detoriate when *t* > 1 for this method as is often the case with numerical schemes such as finite differences.

**Figure 1:**
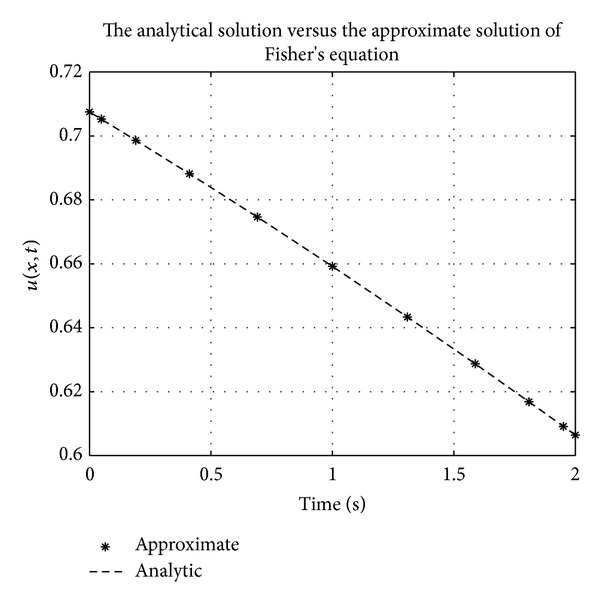
Fishers equation analytical solution graph.

**Figure 2:**
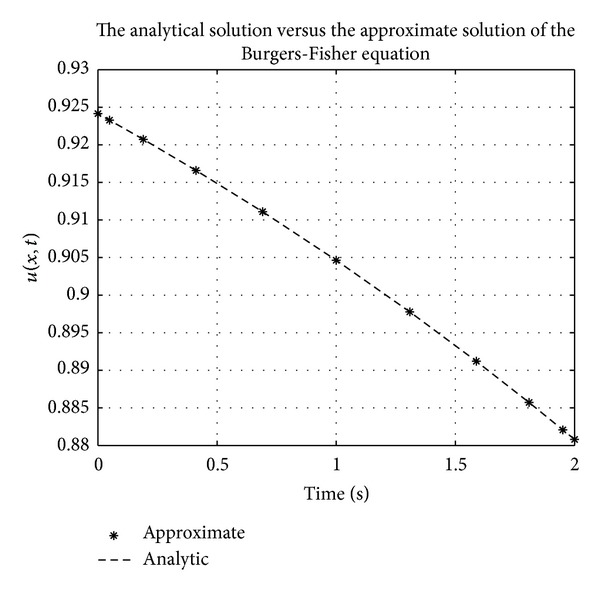
Burger-Fishers equation analytical solution graph.

**Figure 3:**
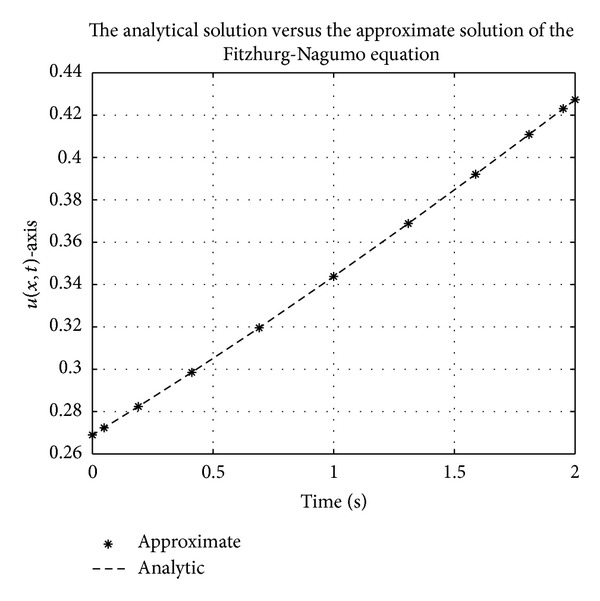
Fitzhugh-Nagumo equation analytical solution graph.

**Figure 4:**
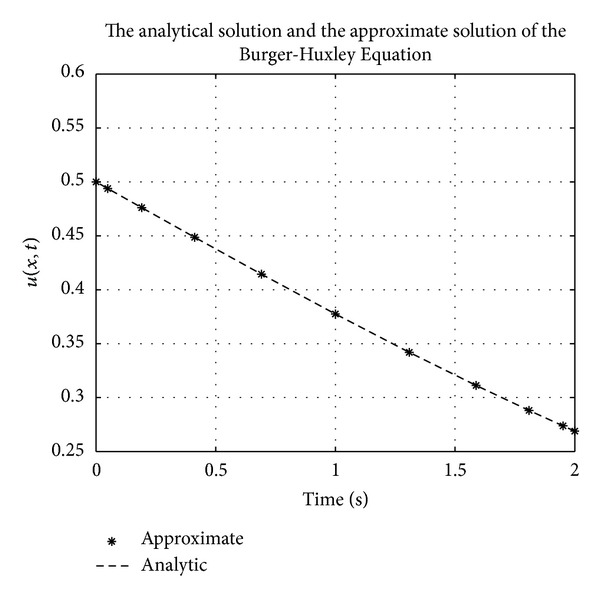
Burgers-Huxley equation analytical solution graph.

**Figure 5:**
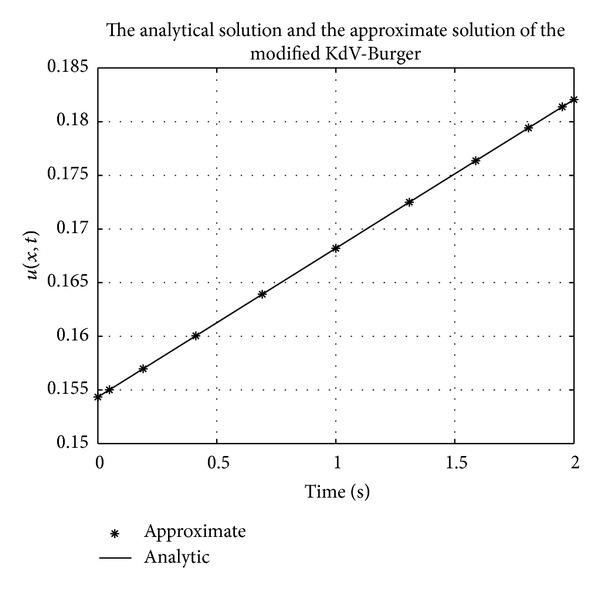
Modified KdV-Burger equation analytical solution graph.

**Figure 6:**
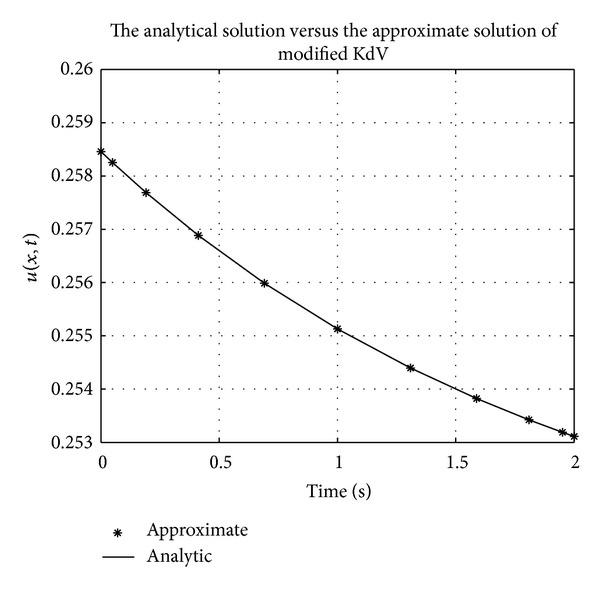
Modified KdV equation analytical solution graph.

Figures 1, 2, 3, 4, 5, and 6 show a comparison of the analytical and approximate solutions of the Fisher equation, Burgers-Fisher equation, Fitzhugh-Nagumo equation, Burgers-Huxley equation, the modified KdV-Burgers equation, and the modified KdV equation, respectively, when *t* = 2. The approximate solutions are in excellent agreement with the analytical solutions, and this demonstrates the accuracy of the algorithm presented in this study.

**Figure 7:**
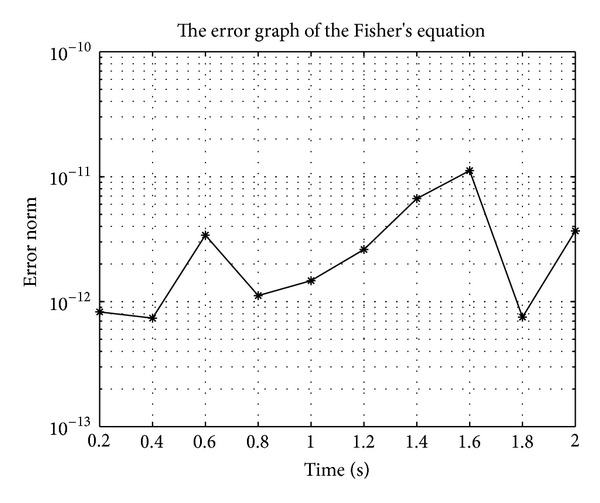
Fishers equation error graph.

**Figure 8:**
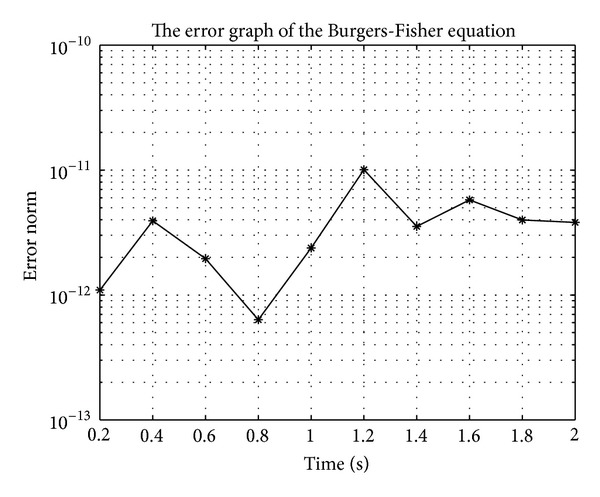
Burger-Fishers equation error graph.

**Figure 9:**
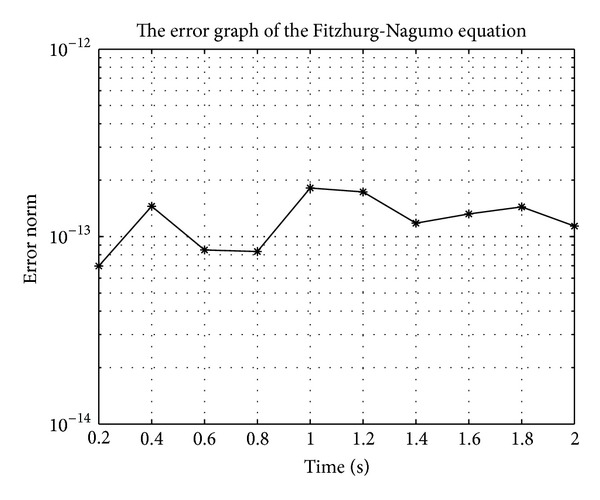
Fitzhugh-Nagumo equation error graph.

**Figure 10:**
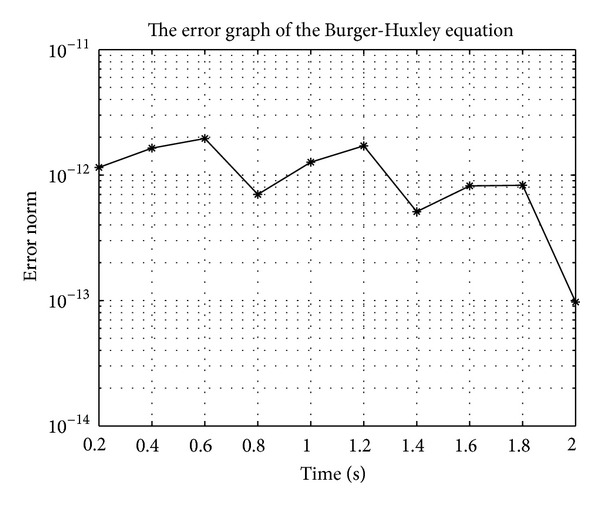
Burgers-Huxley equation error graph.

**Figure 11:**
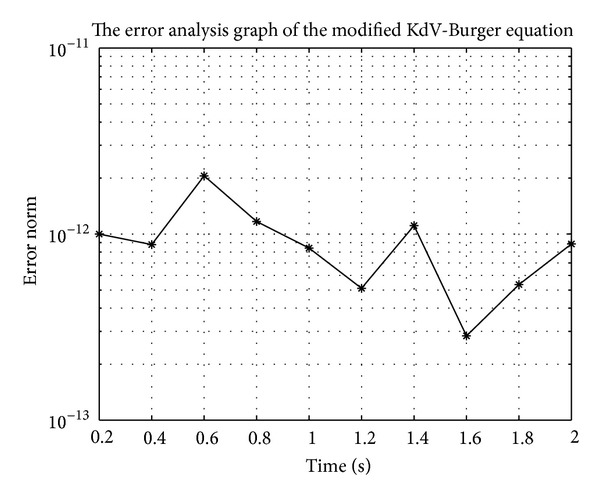
Modified KdV-Burger equation error graph.

**Figure 12:**
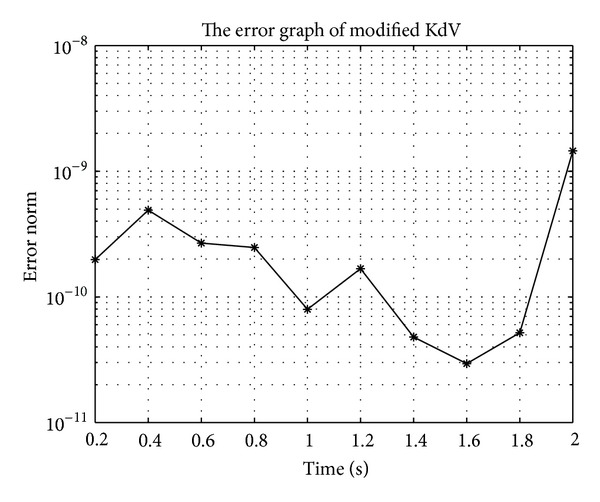
Modified KdV equation error graph.

In Figures 7, 8, 9, 10, 11, and 12, we present error analysis graphs for the Fisher equation, Burgers-Fisher equation, Fitzhugh-Nagumo equation, Burgers-Huxley equation, the modified KdV-Burgers equation, and the modified KdV equation, respectively, when *t* = 2.

**Figure 13:**
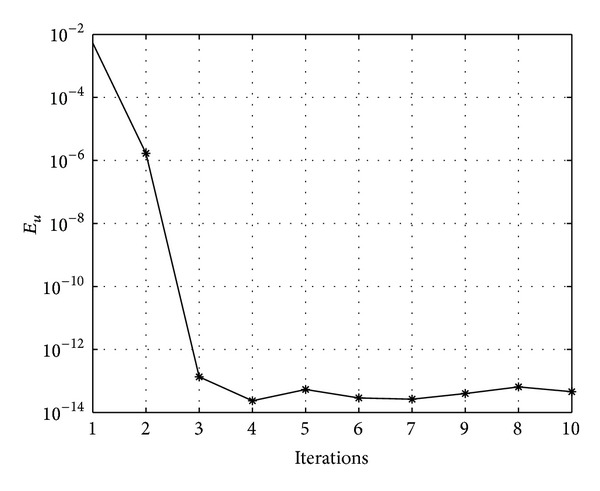
Fishers equation convergence graph.

**Figure 14:**
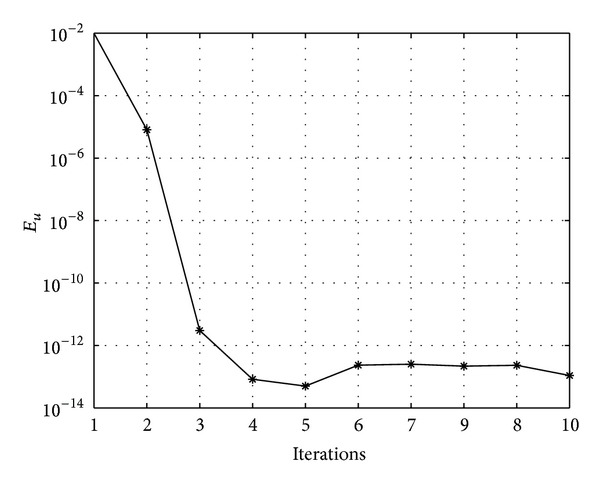
Burger-Fishers equation convergence graph.

**Figure 15:**
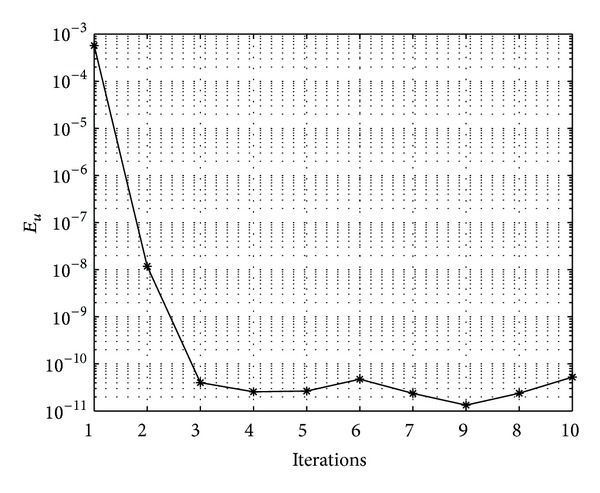
Fitzhugh-Nagumo equation convergence graph.

**Figure 16:**
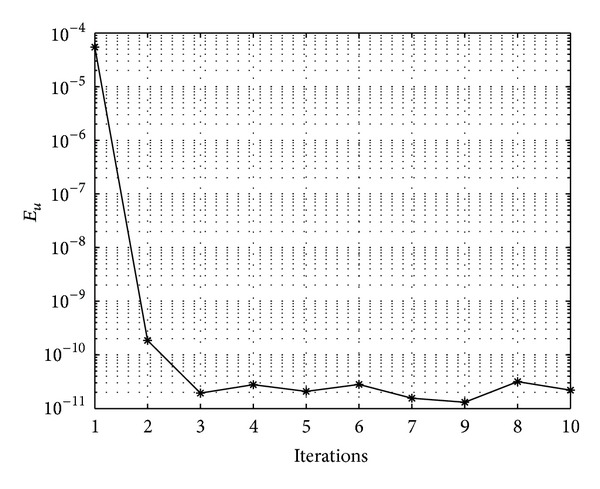
Burgers-Huxley equation convergence graph.

**Figure 17:**
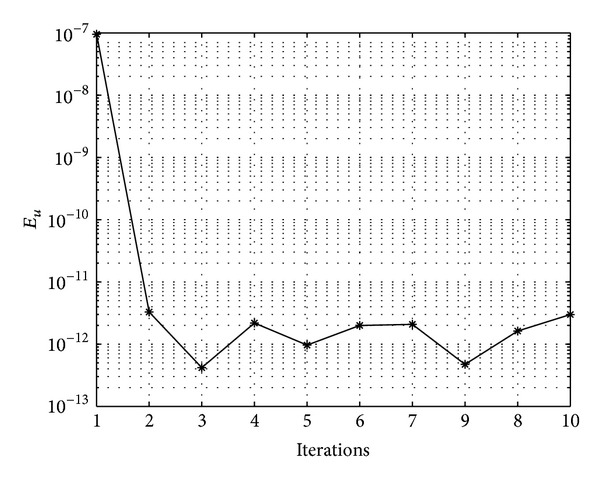
Modified KdV-Burger equation convergence graph.

**Figure 18:**
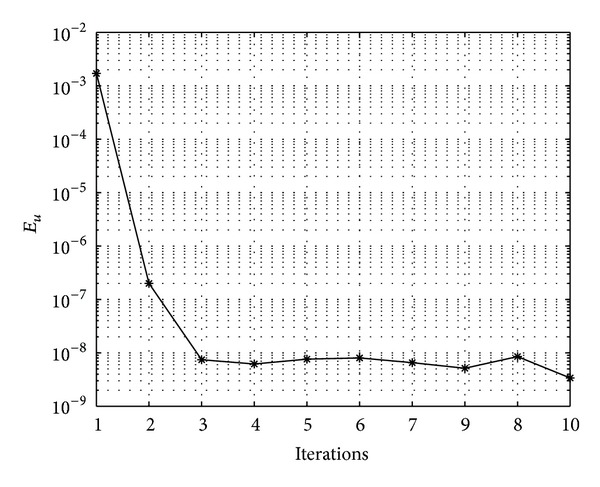
Modified KdV equation convergence graph.

In Figures 13, 14, 15, 16, 17, and 18, convergence analysis graphs for the Fisher equation, Burgers-Fisher equation, Fitzhugh-Nagumo equation, Burgers-Huxley equation, the modified KdV-Burgers equation, and the modified KdV equation, respectively. The figures present a variation of the error norm at a fixed value of time (*t* = 1) with iterations of the BI-SQLM scheme. It can be seen that, in almost all the examples considered, the iteration scheme takes about 3 or 4 iterations to converge fully. Beyond the point where full convergence is reached, error norm levels off and does not improve with an increase in the number of iterations. This plateau level gives an estimate of the maximum error that can be achieved when using the proposed method with a certain number of collocation points. It is worth remarking that the accuracy of the method depends on the number of collocation points in both the *x* and *t* directions. The results from Figures 13–18 clearly demonstrate that the BI-SQLM is accurate.

## 5. Conclusion

This paper has presented a new Chebyshev collocation spectral method for solving general nonlinear evolution partial differential equations. The bivariate interpolated spectral quasilinearisation method (BI-SQLM) was developed by combining elements of the quasilinearisation method and Chebyshev spectral collocation with bivariate Lagrange interpolation. The main goal of the current study was to assess the accuracy, robustness, and effectiveness of the method in solving nonlinear partial differential equations.

Numerical simulations were conducted on the modified KdV-Burger equation, highly nonlinear modified KdV equation, the Fisher equation, Burgers-Fisher equation, Fitzhugh-Nagumo equation, and Burgers-Huxley equation. It is evident from the study that the BI-SQLM gives accurate results in a computationally efficient manner. Further evidence from this study is that the BI-SQLM gives solutions that are uniformly accurate and valid in large intervals of space and time domains. The apparent success of the method can be attributed to the use of the Chebyshev spectral collocation method with bivariate Lagrange interpolation in space and time for differentiating. This work contributes to the existing body of literature on quasilinearisation tools for solving complex nonlinear partial differential equations. Further work needs to be done to establish whether the BI-SQLM can be equally successful in solving coupled systems of equations.

## Figures and Tables

**Table 1:** Maximum errors *E*_*N*_ for Fisher equation when *α* = 1 using *N*_*t*_ = 10.

*t*∖*N*_*x*_	4	6	8	10
0.1	1.986*e* − 008	1.119*e* − 011	7.398*e* − 013	7.171*e* − 013
0.2	3.934*e* − 008	3.121*e* − 011	1.552*e* − 012	1.561*e* − 012
0.3	5.577*e* − 008	4.864*e* − 011	1.004*e* − 012	1.005*e* − 012
0.4	6.997*e* − 008	6.802*e* − 011	7.895*e* − 013	8.124*e* − 013
0.5	8.107*e* − 008	7.971*e* − 011	1.088*e* − 012	1.027*e* − 012
0.6	8.891*e* − 008	8.560*e* − 011	8.805*e* − 013	7.847*e* − 013
0.7	9.344*e* − 008	8.953*e* − 011	6.418*e* − 013	6.463*e* − 013
0.8	9.431*e* − 008	8.759*e* − 011	6.199*e* − 013	6.164*e* − 013
0.9	9.178*e* − 008	8.325*e* − 011	3.978*e* − 013	3.695*e* − 013
1.0	8.787*e* − 008	7.421*e* − 011	7.988*e* − 014	5.596*e* − 014

CPU time (sec)	0.019942	0.025988	0.027756	0.029436

**Table 2:** Maximum errors *E*_*N*_ for the Burgers-Fisher equation when *α* = *γ* = *δ* = 1 using *N*_*t*_ = 10.

*t*∖*N*_*x*_	4	6	8	10
0.1	1.142*e* − 007	1.369*e* − 010	5.891*e* − 012	6.143*e* − 012
0.2	1.178*e* − 007	1.373*e* − 010	9.570*e* − 012	1.013*e* − 011
0.3	1.186*e* − 007	1.479*e* − 010	1.489*e* − 011	1.512*e* − 011
0.4	1.069*e* − 007	9.450*e* − 011	1.703*e* − 011	1.702*e* − 011
0.5	9.030*e* − 008	7.944*e* − 011	5.283*e* − 012	5.736*e* − 012
0.6	6.963*e* − 008	6.618*e* − 011	1.639*e* − 011	1.626*e* − 011
0.7	4.638*e* − 008	1.579*e* − 011	1.362*e* − 011	1.364*e* − 011
0.8	2.457*e* − 008	4.030*e* − 011	3.934*e* − 012	3.852*e* − 012
0.9	2.028*e* − 008	6.006*e* − 011	4.466*e* − 012	4.727*e* − 012
1.0	3.147*e* − 008	7.708*e* − 011	7.757*e* − 013	7.261*e* − 013

CPU Time (sec)	0.010152	0.015387	0.019163	0.021564

**Table 3:** Maximum errors *E*_*N*_ for the Fitzhug-Nagumo equation when *α* = 1 using *N*_*t*_ = 10.

*t*∖*N*_*x*_	4	6	8	10
0.1	5.719*e* − 007	1.196*e* − 009	2.367*e* − 012	9.881*e* − 014
0.2	6.193*e* − 007	1.299*e* − 009	2.761*e* − 012	3.952*e* − 014
0.3	6.662*e* − 007	1.463*e* − 009	3.259*e* − 012	8.216*e* − 014
0.4	6.779*e* − 007	1.448*e* − 009	3.341*e* − 012	8.094*e* − 014
0.5	6.920*e* − 007	1.526*e* − 009	3.587*e* − 012	5.063*e* − 014
0.6	7.019*e* − 007	1.573*e* − 009	3.729*e* − 012	3.775*e* − 014
0.7	6.933*e* − 007	1.516*e* − 009	3.660*e* − 012	8.915*e* − 014
0.8	6.828*e* − 007	81.535*e* − 009	3.635*e* − 012	7.594*e* − 014
0.9	6.765*e* − 007	1.528*e* − 009	3.519*e* − 012	3.242*e* − 013
1.0	6.687*e* − 007	1.490*e* − 009	3.405*e* − 012	1.688*e* − 013

CPU time (sec)	0.024281	0.024901	0.026810	0.032389

**Table 4:** Maximum errors *E*_*N*_ for the Burger-Huxley equation when *γ* = 0.75, *β* = 1, and *N*_*t*_ = 10.

*t*∖*N*_*x*_	4	6	8	10
0.1	2.217*e* − 006	8.482*e* − 009	2.166*e* − 011	7.822*e* − 014
0.2	2.596*e* − 006	9.369*e* − 009	2.536*e* − 011	1.184*e* − 013
0.3	2.859*e* − 006	1.073*e* − 008	3.201*e* − 011	1.049*e* − 013
0.4	3.001*e* − 006	1.112*e* − 008	3.652*e* − 011	9.426*e* − 014
0.5	3.137*e* − 006	1.213*e* − 008	4.262*e* − 011	1.510*e* − 013
0.6	3.270*e* − 006	1.311*e* − 008	4.842*e* − 011	2.127*e* − 013
0.7	3.367*e* − 006	1.359*e* − 008	5.289*e* − 011	1.230*e* − 013
0.8	3.467*e* − 006	1.438*e* − 008	5.803*e* − 011	1.549*e* − 013
0.9	3.562*e* − 006	1.504*e* − 008	6.260*e* − 011	3.063*e* − 013
1.0	3.640*e* − 006	1.559*e* − 008	6.674*e* − 011	2.951*e* − 013

CPU time (sec)	0.023822	0.024901	0.02685	0.032806

**Table 5:** Maximum errors *E*_*N*_ for the modified KdV-Burgers equation, with *N*_*t*_ = 10.

*t*∖*N*_*x*_	4	6	8	10
0.1	1.803*e* − 007	3.419*e* − 010	4.449*e* − 013	1.572*e* − 013
0.2	2.614*e* − 007	4.347*e* − 010	5.049*e* − 013	5.992*e* − 014
0.3	2.717*e* − 007	4.677*e* − 010	5.532*e* − 013	8.128*e* − 013
0.4	2.009*e* − 007	3.663*e* − 010	4.771*e* − 013	6.158*e* − 013
0.5	2.580*e* − 007	4.410*e* − 010	7.518*e* − 013	2.555*e* − 013
0.6	2.653*e* − 007	4.606*e* − 010	8.738*e* − 013	5.756*e* − 013
0.7	2.248*e* − 007	4.039*e* − 010	6.210*e* − 013	2.393*e* − 013
0.8	2.572*e* − 007	4.476*e* − 010	5.432*e* − 013	6.812*e* − 013
0.9	2.436*e* − 007	4.351*e* − 010	6.111*e* − 013	6.287*e* − 013
1.0	8.275*e* − 008	3.721*e* − 010	7.569*e* − 013	1.087*e* − 007

CPU time (sec)	0.015646	0.021226	0.030159	0.035675

**Table 6:** Maximum errors *E*_*N*_ for the highly nonlinear modified KdV equation, with *N*_*t*_ = 10.

*t*∖*N*_*x*_	4	6	8	10
0.1	7.788*e* − 005	3.553*e* − 007	7.601*e* − 010	2.080*e* − 010
0.2	1.153*e* − 004	4.000*e* − 007	5.684*e* − 010	1.189*e* − 010
0.3	1.011*e* − 004	3.739*e* − 007	4.471*e* − 010	4.503*e* − 010
0.4	3.926*e* − 005	1.785*e* − 007	6.544*e* − 010	4.987*e* − 010
0.5	6.727*e* − 005	2.342*e* − 007	2.638*e* − 010	1.528*e* − 010
0.6	6.065*e* − 005	2.207*e* − 007	4.565*e* − 010	4.568*e* − 010
0.7	2.511*e* − 005	1.105*e* − 007	4.749*e* − 010	3.748*e* − 010
0.8	4.074*e* − 005	1.427*e* − 007	1.062*e* − 010	1.604*e* − 010
0.9	2.386*e* − 005	1.018*e* − 007	2.343*e* − 010	8.114*e* − 011
1.0	1.440*e* − 006	7.256*e* − 008	1.436*e* − 009	1.513*e* − 011

CPU time (sec)	0.020609	0.021241	0.030617	0.032816

**Table 7:** Maximum errors *E*_*N*_ for Fisher equation when *α* = 1 using *N*_*t*_ = 10.

*t*∖*N*_*x*_	4	6	8	10
0.2	1.119*e* − 011	7.398*e* − 013	8.266*e* − 013	3.808*e* − 014
0.4	3.121*e* − 011	1.552*e* − 012	7.378*e* − 013	3.780*e* − 014
0.6	4.864*e* − 011	1.004*e* − 012	3.402*e* − 012	7.283*e* − 014
0.8	6.802*e* − 011	7.895*e* − 013	1.118*e* − 012	3.714*e* − 014
1.0	7.971*e* − 011	1.088*e* − 012	1.473*e* − 012	1.691*e* − 013
1.2	8.560*e* − 011	8.805*e* − 013	2.611*e* − 012	3.119*e* − 013
1.4	8.953*e* − 011	6.418*e* − 013	6.671*e* − 012	1.796*e* − 013
1.6	8.759*e* − 011	6.199*e* − 013	1.118*e* − 011	1.097*e* − 013
1.8	8.325*e* − 011	3.978*e* − 013	7.515*e* − 013	6.273*e* − 014
2.0	7.421*e* − 011	7.988*e* − 014	3.682*e* − 012	2.311*e* − 013

CPU time (sec)	0.013542	0.022967	0.023792	0.024758

**Table 8:** Maximum errors *E*_*N*_ for the Burgers-Fisher equation when *α* = 1 using *N*_*t*_ = 10.

*t*∖*N*_*x*_	4	6	8	10
0.2	1.223*e* − 007	1.400*e* − 008	1.402*e* − 008	1.094*e* − 012
0.4	1.145*e* − 007	1.919*e* − 008	1.918*e* − 008	3.919*e* − 012
0.6	9.192*e* − 008	2.082*e* − 008	2.085*e* − 008	1.953*e* − 012
0.8	2.293*e* − 008	1.793*e* − 008	1.793*e* − 008	6.340*e* − 013
1.0	2.395*e* − 008	1.337*e* − 008	1.339*e* − 008	2.381*e* − 012
1.2	5.778*e* − 008	1.954*e* − 008	1.930*e* − 008	1.005*e* − 011
1.4	6.045*e* − 008	1.620*e* − 008	1.620*e* − 008	3.535*e* − 012
1.6	5.244*e* − 008	7.218*e* − 009	7.345*e* − 009	5.765*e* − 012
1.8	4.395*e* − 008	6.828*e* − 009	6.784*e* − 009	3.983*e* − 012
2.0	2.944*e* − 008	9.406*e* − 010	8.820*e* − 010	3.812*e* − 012

CPU time (sec)	0.019942	0.025988	0.027756	0.029436

**Table 9:** Maximum errors *E*_*N*_ for the Fitzhugh-Nagumo equation when *α* = 1 using *N*_*t*_ = 10.

*t*∖*N*_*x*_	4	6	8	10
0.2	6.326*e* − 007	1.311*e* − 009	2.886*e* − 012	1.131*e* − 012
0.4	6.721*e* − 007	1.467*e* − 009	3.310*e* − 012	1.564*e* − 012
0.6	7.140*e* − 007	1.602*e* − 009	3.617*e* − 012	1.936*e* − 012
0.8	6.730*e* − 007	1.496*e* − 009	4.707*e* − 012	1.196*e* − 012
1.0	6.660*e* − 007	1.487*e* − 009	3.675*e* − 012	1.264*e* − 012
1.2	6.449*e* − 007	1.366*e* − 009	1.897*e* − 012	1.727*e* − 012
1.4	5.690*e* − 007	1.083*e* − 009	2.972*e* − 012	1.200*e* − 012
1.6	4.931*e* − 007	8.010*e* − 010	1.519*e* − 012	8.590*e* − 013
1.8	3.986*e* − 007	4.658*e* − 010	1.068*e* − 012	6.790*e* − 013
2.0	2.904*e* − 007	2.968*e* − 010	1.592*e* − 012	1.770*e* − 013

CPU time (sec)	0.041048	0.049629	0.055008	0.053863

**Table 10:** Maximum errors *E*_*N*_ for the Burgers-Huxley equation when *γ* = 0.5, *β* = 1, and *N*_*t*_ = 10.

*t*∖*N*_*x*_	4	6	8	10
0.2	2.866*e* − 006	1.119*e* − 008	3.670*e* − 011	1.150*e* − 012
0.4	3.401*e* − 006	1.420*e* − 008	5.744*e* − 011	1.638*e* − 012
0.6	3.814*e* − 006	1.687*e* − 008	7.426*e* − 011	1.958*e* − 012
0.8	3.915*e* − 006	1.729*e* − 008	8.171*e* − 011	7.002*e* − 013
1.0	3.938*e* − 006	1.738*e* − 008	8.157*e* − 011	1.267*e* − 012
1.2	3.808*e* − 006	1.624*e* − 008	7.687*e* − 011	1.710*e* − 012
1.4	3.456*e* − 006	1.527*e* − 008	6.965*e* − 011	5.109*e* − 013
1.6	3.230*e* − 006	1.349*e* − 008	5.535*e* − 011	8.203*e* − 013
1.8	2.925*e* − 006	1.078*e* − 008	3.598*e* − 011	8.294*e* − 013
2.0	2.497*e* − 006	7.505*e* − 009	2.265*e* − 011	9.726*e* − 014

CPU time (sec)	0.023822	0.024901	0.02685	0.032806

**Table 11:** Maximum errors *E*_*N*_ for the modified KdV-Burgers equation, with *N*_*t*_ = 10.

*t*∖*N*_*x*_	4	6	8	10
0.2	2.137*e* − 007	3.820*e* − 010	4.846*e* − 013	9.998*e* − 013
0.4	2.480*e* − 007	4.267*e* − 010	5.596*e* − 013	8.775*e* − 013
0.6	2.691*e* − 007	4.676*e* − 010	6.565*e* − 013	2.054*e* − 012
0.8	2.214*e* − 007	3.979*e* − 010	8.776*e* − 013	1.168*e* − 012
1.0	2.538*e* − 007	4.463*e* − 010	9.650*e* − 013	8.410*e* − 013
1.2	2.650*e* − 007	4.680*e* − 010	7.450*e* − 013	5.113*e* − 013
1.4	2.383*e* − 007	4.296*e* − 010	7.500*e* − 013	1.110*e* − 012
1.6	2.568*e* − 007	4.572*e* − 010	9.704*e* − 013	2.837*e* − 013
1.8	2.520*e* − 007	4.529*e* − 010	7.443*e* − 013	5.353*e* − 013
2.0	2.370*e* − 007	4.438*e* − 010	2.719*e* − 013	8.849*e* − 013

CPU time (sec)	0.062066	0.081646	0.080718	0.10775

**Table 12:** Maximum errors *E*_*N*_ for the highly nonlinear modified KdV equation, with *N*_*t*_ = 10.

*t*∖*N*_*x*_	4	6	8	10
0.2	1.986*e* − 008	1.119*e* − 011	7.398*e* − 013	7.171*e* − 013
0.4	8.010*e* − 005	3.577*e* − 007	3.902*e* − 008	1.979*e* − 010
0.6	7.235*e* − 005	2.549*e* − 007	2.016*e* − 008	4.899*e* − 010
0.8	6.284*e* − 005	1.663*e* − 007	1.155*e* − 007	2.679*e* − 010
1.0	1.642*e* − 005	1.620*e* − 007	1.243*e* − 007	2.474*e* − 010
1.2	2.753*e* − 005	1.073*e* − 007	1.073*e* − 007	1.679*e* − 010
1.4	3.738*e* − 006	8.971*e* − 008	8.598*e* − 008	4.788*e* − 011
1.6	1.223*e* − 005	2.153*e* − 008	2.503*e* − 008	2.941*e* − 011
1.8	5.836*e* − 006	2.986*e* − 008	9.127*e* − 009	5.177*e* − 011
2.0	9.310*e* − 006	6.548*e* − 008	7.277*e* − 008	1.453*e* − 009

CPU time (sec)	0.020609	0.021241	0.030617	0.032816
